# Heteroclinic cycling and extinction in May–Leonard models with demographic stochasticity

**DOI:** 10.1007/s00285-022-01859-4

**Published:** 2023-01-13

**Authors:** Nicholas W. Barendregt, Peter J. Thomas

**Affiliations:** 1 Department of Applied Mathematics, University of Colorado Boulder, 1111 Engineering Center, ECOT 225, 526 UCB, Boulder, CO 80309 USA; 2 Department of Mathematics, Applied Mathematics, and Statistics; Department of Biology; Department of Cognative Science; Department of Data and Computer Science; Department of Electrical, Control and Systems Engineering, Case Western Reserve University, Yost Hall, 2049 Martin Luther King Jr. Drive, Cleveland, OH 44106 USA

**Keywords:** Stochastic modeling, Heteroclinic cycling, Theoretical ecology, Computational neuroscience, 92B05, 37C29, 60J27, 60J22

## Abstract

May and Leonard (SIAM J Appl Math 29:243–253, 1975) introduced a three-species Lotka–Volterra type population model that exhibits heteroclinic cycling. Rather than producing a periodic limit cycle, the trajectory takes longer and longer to complete each “cycle”, passing closer and closer to unstable fixed points in which one population dominates and the others approach zero. Aperiodic heteroclinic dynamics have subsequently been studied in ecological systems (side-blotched lizards; colicinogenic *Escherichia coli*), in the immune system, in neural information processing models (“winnerless competition”), and in models of neural central pattern generators. Yet as May and Leonard observed “Biologically, the behavior (produced by the model) is nonsense. Once it is conceded that the variables represent animals, and therefore cannot fall below unity, it is clear that the system will, after a few cycles, converge on some single population, extinguishing the other two.” Here, we explore different ways of introducing discrete stochastic dynamics based on May and Leonard’s ODE model, with application to ecological population dynamics, and to a neuromotor central pattern generator system. We study examples of several quantitatively distinct asymptotic behaviors, including total extinction of all species, extinction to a single species, and persistent cyclic dominance with finite mean cycle length.

## Introduction

Following Lotka (1925) and Volterra (1926), May and Leonard (1975) introduced a model generalizing Lotka-Volterra dynamics for a system of three species:

1 $$\begin{aligned} \begin{aligned} \frac{dn_1}{dt}={}&n_1\left( 1-n_1-\alpha n_2-\beta n_3\right) ,\\ \frac{dn_2}{dt}={}&n_2\left( 1-\beta n_1-n_2-\alpha n_3\right) ,\\ \frac{dn_3}{dt}={}&n_3\left( 1-\alpha n_1-\beta n_2-n_3\right) . \end{aligned} \end{aligned}$$

In Eq. (1) $n_i$ represents the population of species *i*, and the constants $\alpha \ge 0$ and $\beta \ge 0$ represent the strengths of competitive interactions. The model exhibits different types of coexistence of solutions for different choices of $\alpha$ and $\beta$. When $\alpha +\beta =2$, the system converges to a periodic orbit contained in the plane $n_1+n_2+n_3=1$. This solution can be interpreted as the direct extension of Lotka-Volterra to three species, where each species’ population oscillates with finite period. However, when $\alpha +\beta >2$ and either $\alpha < 1$ or $\beta < 1$, the system undergoes heteroclinic cycling, with the duration of each cycle increasing as time progresses. In this regime, each species’ population becomes closer to zero with each cycle, and spends a longer fraction of each cycle in this near-extinction state.

Heteroclinic cycling models such as Eq. (1) and their variants have frequently served as models for “rock-paper-scissors”-type population dynamics in which populations take turns as the dominant species before being pushed out in favor of a more competitive population. Sinervo and Lively (1996) found that a species of side-blotched lizards exhibits rock-paper-scissors competition: orange aggressive lizards beat out less-aggressive blue lizards for mates, yellow “sneaker” lizards invade the larger orange lizard territory to steal mates, and blue lizards beat out the sneakers for mates. Kerr et al. (2002) observed a similar behavior in colicinogenic *E. coli*: a toxin-producing strain kills a susceptible population, a toxin-resistant population grows faster than the toxin-producing population, and the susceptible population grows faster than the resistant population. Rock-paper-scissors models have also been considered subject to environmental stochasticity (Hening and Li 2021). In computational neuroscience, heteroclinic cycling has been proposed as an alternative to classic “winner-take-all” models for neural networks. Rabinovich et al. (2001; 2008) suggested that the activity of olfactory neurons when encoding stimuli can be projected onto a heteroclinic cycle and called the behavior “winnerless competition.” Varona et al. (2002) theorized that high-dimensional heteroclinic systems leading to chaotic dynamics might underlie the apparently random search behavior during hunting in the mollusc *Clione*. Shaw et al. (2015) and Lyttle et al. (2017) constructed a model capable of transitioning between limit-cycling and heteroclinic-cycling behaviors to represent a neuromotor central pattern generator (CPG) in *Aplysia californica* (see also Park et al. (2018)). While more detailed models for the *Aplysia* feeding system have since been developed (Webster Wood et al. 2020), the simplicity of the three-component SLG (Shaw-Lyttle-Gill) model makes it an attractive target for analysis.

Despite their popularity, heteroclinic cycling models of biological populations, when formulated as systems of ordinary differential equations, suffer a fundamental flaw. Indeed, in their original paper, May and Leonard noted a significant drawback of their model’s ability to describe population dynamics. They observed that, while heteroclinic cycling continues indefinitely, real biological populations “cannot fall below unity, [and] it is clear that the system will, after a few cycles, converge on some single population, extinguishing the other two” (May and Leonard 1975). This discrepancy arises from *demographic stochasticity*, that is, noise arising from small discrete population numbers (copy number noise), that is inherent in systems where populations take on discrete integer values.

In light of May and Leonard’s observation, one might expect that a stochastic system undergoing heteroclinic cycling would necessarily exhibit population extinctions. However, as is well known, the mapping from a given ODE model to a stochastic model having matching mean-field dynamics is not unique. For example, Allen (2010) noted that for a logistic birth-death process, there are an infinite number of per capita birth and death rates that yield the same mean-field logistic growth. Xue and Goldenfeld (2017) found that modeling plankton ecosystems using stochastic versions of the “kill-the-winner” model resulted in extinction events, while the mean-field model had stable coexistence of all species. And Strang et al. (2019) explored the paradox that stochastic models with the Allee effect, which reduces per-capita growth rate for small population size, can have longer persistence than models without the effect. The ambiguity intrinsic to stochastic extensions of ODE systems is not confined to ecological models. A series of papers have debated the most appropriate way to extend the deterministic Hodgkin-Huxley equations to incorporate the effects of random gating of ion channels in neural dynamics (Fox and Lu 1994; Goldwyn et al. 2011; Goldwyn and Shea-Brown 2011; Orio and Soudry 2012; Anderson et al. 2015a; Pu and Thomas 2020, 2021). At the level of large-scale neural circuits, several distinct stochastic generalizations have been proposed that coincide with the classical deterministic Wilson-Cowan neural field equations in the mean-field limit (Bressloff 2010; Benayoun et al. 2010; Faugeras and Inglis 2015; Cowan et al. 2016; De Candia et al. 2021).

Generally speaking, when considering the effects of stochastic fluctuations in a biological system, one may adopt a “bottom-up” or a “top-down” approach. In the bottom-up or first-principles approach, one tries to specify the system components and interactions relevant to the phenomena of interest, and construct an appropriate Markov process capturing the structure and dynamics of the system. In the examples we discussed above, such as understanding the effects of the random gating of ion channels on the dynamics of conductance-based neural systems in Hodgkin-Huxley or Morris-Lecar system (Thomas and Lindner 2014; Pu and Thomas 2020, 2021), or studying the interaction of demographic stochasticity with the Allee effect in a population model (Strang et al. 2019), a bottom-up approach has been highly successful. However, the first-principles approach has its limitations, as it requires specification of all relevant system components and their interactions. In complex systems, such as neurological dynamics in the brain, it is unrealistic to try and fully describe all interactions between cells.

Historically, mathematical biology has relied on ODE models to study interacting populations, even though populations are inherently composed of discrete individuals. This approach goes back to the work of work of Malthus and Verhulst (for a single population) and, as we mentioned previously, Lotka and Volterra (for two interacting populations). Markov chains had been described in the first decade of the 1900s; Lotka and Volterra might have formulated their predatory-prey models in a discrete-state stochastic framework. But the deterministic ODE framework sufficed for investigating the salient system behaviors, such as population oscillations, in relatively simpler terms. Similarly, the Wilson-Cowan equations use Lotka-Volterra type dynamics to describe some of the complex behaviors of interacting neural populations, even though the brain is a highly stochastic system composed of discrete individual neurons producing discrete individual voltage spikes. As we have already mentioned, many groups have sought to develop stochastic representations of neural dynamics constrained so that the mean-field behavior of the stochastic equations is consistent with the Wilson–Cowan equations. Thus, in the attempt to add more realism to models of neural dynamics, many mathematical neuroscientists use the deterministic Wilson–Cowan equations as a heuristic guide and starting point, rather than setting it aside and beginning from scratch and trying to build a model “from first principles.” Here, we take a similar approach to studying the May–Leonard system.

First, we consider two alternative stochastic models, each based on a birth-death formalism consistent with Eq. (1). By formulating the discrete master equation (Gardiner 2009) and leveraging complex-balanced equilibrium results from chemical kinetics (Anderson and Kurtz 2015), we prove that each alternative results in a qualitatively different stationary distribution. We confirm these findings numerically. We then propose a modified May–Leonard system inspired by a neuromotor CPG model from Lyttle et al. (2017). Using the same birth-death formalism, we construct a stochastic implementation of this new model that not only avoids extinction events, but also maintains a finite mean cycle length. We numerically investigate how the mean cycle length depends on model parameters and examine its asymptotic behaviors in the both the large and small system size limits. Taken together, these results illustrate the rich variety of behaviors that may be obtained from different stochastic generalizations of May and Leonard’s original deterministic heteroclinic cycling model.

## Mean-field formulations of heteroclinic cycling

In this section, we begin by fully specifying the mean-field dynamics of the May–Leonard systems we will study so that we have a solid foundation on which to build our stochastic models. For a general system of *m* species following deterministic Lotka-Volterra interactions, species *i* has the governing equation

2 $$\begin{aligned} \frac{dn_i}{dt}=r_in_i\left( 1-\sum _{j=1}^{m}k_{ij}n_j\right) +f_i(t). \end{aligned}$$

In Eq. (2), $n_i$ is the population size of species $i\in \{1,\dots ,m\}$, $r_i$ is the intrinsic growth rate of species *i*, $k_{ij}$ represents the strength the competitive effect of species *j* on species *i*, and $f_i(t)$ is a nonhomogeneous forcing function that can represent immigration, harvesting, etc. of species *i*. We will use Eq. (2) to construct three versions of May and Leonard’s heteroclinic cycling model. For the duration of the paper we will restrict our attention to three interacting species ($m=3$), assume that each species has the same intrinsic growth rate $r_1=r_2=r_3=r$ and forcing function $f_1=f_2=f_3=f$, and enforce that competition rates have the same cyclic symmetry as the May–Leonard system, so that $k_{12}=k_{23}=k_{31}=k_{i,i+1}$, $k_{13}=k_{21}=k_{32}=k_{i,i+2}$ and $k_{11}=k_{22}=k_{33}=k_{ii}$, where indicial addition is taken cyclically. Note that by setting $m=3$, $r=1$, $k_{i,i+1}=\alpha$, $k_{i,i+2}=\beta$, $k_{ii}=1$, and $f=0$, we recover Eq. (1).

The first two models we consider will be direct analogues of Eq. (1). As is the case in May and Leonard’s original system, both models will obey mass-action kinetics, with implications that we discuss below. We begin with a “general variance” or “GV model.” In this model, the intrinsic growth rate *r* reflects the combined effects of a per capita birth rate $b>0$ and a per capita death rate $d>0$, chosen so that $r=b-d>0$. The terminology “general variance" reflects the fact that the variance of the population growth over short times $\Delta t$ scales as $(b+d)\Delta t+o(\Delta t)$. Thus for a given value of *r*, we can obtain arbitrarily large variance in the population growth process by increasing both *b* and *d*. Following the language of Van Kampen (1992) and Gardiner (2009), we introduce a system size parameter $\Omega$ (representing the single-species carrying capacity). We consider the $n_i$ of Eq. (2) as intensive variables and define $N_i=\Omega n_i$ as extensive variables for the number of individuals in the *i*-th species. The resulting mean-field equations for the GV model may be written as:

3 $$\begin{aligned} \begin{aligned} \frac{dN_1}{dt} ={}&N_1\left[ (b-d)-\frac{N_1}{\Omega }-\frac{\alpha }{\Omega } N_2-\frac{\beta }{\Omega } N_3\right] , \\ \frac{dN_2}{dt} ={}&N_2\left[ (b-d)-\frac{\beta }{\Omega } N_1-\frac{N_2}{\Omega }-\frac{\alpha }{\Omega } N_3\right] , \\ \frac{dN_3}{dt} ={}&N_3\left[ (b-d)-\frac{\alpha }{\Omega } N_1-\frac{\beta }{\Omega } N_2-\frac{N_3}{\Omega }\right] . \end{aligned} \end{aligned}$$

For notational clarity, we write the birth and death rates separately; in the stochastic model each will parametrize a separate stochastic reaction term (see Sect. 3.1). Note that when $N_2=N_3=0$, $N_1$ follows logistic growth with carrying capacity $\Omega$ and low-density growth rate $(b-d)$. Additionally, Eq. (3) highlights that tracking extensive variables rather than intensive variables is equivalent to a change of units and does not influence the dynamics of the deterministic system. However, when constructing our stochastic systems in Sect. 3, we will see that it is easier to start from an ODE system of extensive variables.

The second model we consider may be seen as a special case of the GV model, given by setting the intrinsic growth rate $r=b$ and the per capita death rate $d=0$. While this restriction may seem nonphysical, it may be a good approximation of some biological systems. For example, some bacterial populations survive exposure to antibiotics by entering a “persistent state” for which the mortality rate is effectively zero (see Gerdes and Maisonneuve (2012); Browning et al. (2021) for details). As noted above, the variance of the population growth over short times is proportional to $b+d$. Therefore, for a fixed *r*, the assumption $d=0$ gives the *minimum variance model*, which we call the “minimal model.” Its mean-field equations are:

4 $$\begin{aligned} \begin{aligned} \frac{dN_1}{dt} ={}&N_1\left[ r-\frac{N_1}{\Omega }-\frac{\alpha }{\Omega } N_2-\frac{\beta }{\Omega } N_3\right] , \\ \frac{dN_2}{dt} ={}&N_2\left[ r-\frac{\beta }{\Omega } N_1-\frac{N_2}{\Omega }-\frac{\alpha }{\Omega } N_3\right] , \\ \frac{dN_3}{dt} ={}&N_3\left[ r-\frac{\alpha }{\Omega } N_1-\frac{\beta }{\Omega } N_2-\frac{N_3}{\Omega }\right] . \end{aligned} \end{aligned}$$

In Eq. (4), we replaced the individual birth and death rates from the GV model with the net growth rate *r*. Again note that if $b-d=r$ from Eq. (3), the GV and minimal models are equivalent at the level of mean-field equations, and we recover Eq. (1) by taking $\Omega =b-d=r\equiv 1$. However, in the stochastic implementation of the minimal model, eliminating the death process qualitatively changes the long-time asymptotic behavior (see Sect. 3.2).

As a third stochastic variation on the May–Leonard model, we explore the effect of the nonhomogeneous term $f\ne 0$. This variation is motivated by heteroclinic cycling models of neural CPGs. For example, Shaw et al. (2015) and Lyttle et al. (2017) proposed a model for a CPG driving feeding movements in the marine mollusk *Aplysia californica* that comprises three pools of motor neurons, coupled by reciprocal inhibition and driven by endogenous activation. Each neural pool has an activation variable, $n_i$, $i\in \{0,1,2\}$ that represents the fraction of active neurons in the *i*-th pool, ranging from $n_i=0$ (inactive) to $n_i=1$ (fully active). Following the original model proposed by Shaw et al. (2015) and Lyttle et al. (2017), we assume the interactions between neural pools satisfy May–Leonard type competitive dynamics. To study the effects of demographic stochasticity, we start by mirroring the formalism of the Wilson-Cowan equations (Wilson and Cowan 1972, 1973) and interpret the $n_i$ as intensive variables representing the fraction of active neurons in *i*-th pool. We introduce a system size $\Omega$, corresponding to the number of cells in each pool, and write $N_i=\Omega n_i$ as extensive variables, representing the integer number of active cells. We thus obtain our third mean-field model, which we call the “three-pool model:”

5 $$\begin{aligned} \begin{aligned} \frac{dN_0}{dt}={}&\frac{1}{\tau }\left[ N_0\left( 1-\frac{N_0}{\Omega }-\frac{\gamma }{\Omega } N_1\right) +\mu \left( \Omega -N_0\right) \right] ,\\ \frac{dN_1}{dt}={}&\frac{1}{\tau }\left[ N_1\left( 1-\frac{N_1}{\Omega }-\frac{\gamma }{\Omega } N_2\right) +\mu \left( \Omega -N_1\right) \right] ,\\ \frac{dN_2}{dt}={}&\frac{1}{\tau }\left[ N_2\left( 1-\frac{N_2}{\Omega }-\frac{\gamma }{\Omega } N_0\right) +\mu \left( \Omega -N_2\right) \right] . \end{aligned} \end{aligned}$$

Note that Eq. (5) can be obtained from Eq. (2) by taking $r=\frac{1-\mu }{\tau }$, $k_{ii}=\frac{1}{\tau \Omega }$, $k_{i,i+1}=\frac{\gamma }{\tau \Omega }$, $k_{i,i+2}=0$, and $f=\frac{\mu \Omega }{\tau }$. In Eq. (5), $\tau$ is a time constant, $\gamma$ is the strength of inhibition, and $\mu$ governs the rate of endogenous activation. This activation parameter $\mu$ represents intrinsic sources of excitation, whether from ongoing network activity, slow endogenous excitatory currents, or neuromodulatory effects, that cause cells to activate spontaneously. This endogenous activation provides an additional source of stochasticity in our model. In this model, the total number of cells in each neural pool is conserved, with transitions representing changes of activation state rather than “births" or “deaths". In contrast to the ecological models, Eqs. (3) and (4), where population sizes are unbounded, in the neural pool model the population state-space is finite. The endogenous activation term $\mu \ll 1$ was introduced by Shaw et al. (2015) as a means of regulating the sensitivity of the neural activity, by steering trajectories away from the saddle points of the heteroclinic system. Here we define the endogenous activation term somewhat differently from their original formulation, in order to enforce zero flux conditions on the boundaries of our space, which in turn allows us to construct a well-defined stochastic model (see Sect. 5). As in the GV and minimal models, the three-pool model obeys mass-action kinetics; to see this, define $M_i=\Omega -N_i$ to be the number of inactive neurons in the *i*-th pool. We may then rewrite Eq. (5) as:

$$\begin{aligned} \begin{aligned} \frac{dN_0}{dt}={}&\frac{1}{\tau }\left[ \frac{1}{\Omega }N_0M_0-\frac{\gamma }{\Omega }N_0N_1+\mu M_0\right] ,\\ \frac{dN_1}{dt}={}&\frac{1}{\tau }\left[ \frac{1}{\Omega }N_1M_1-\frac{\gamma }{\Omega }N_1N_2+\mu M_1\right] ,\\ \frac{dN_2}{dt}={}&\frac{1}{\tau }\left[ \frac{1}{\Omega }N_2M_2-\frac{\gamma }{\Omega }N_2N_0+\mu M_2\right] . \end{aligned} \end{aligned}$$

**Fig. 1:**
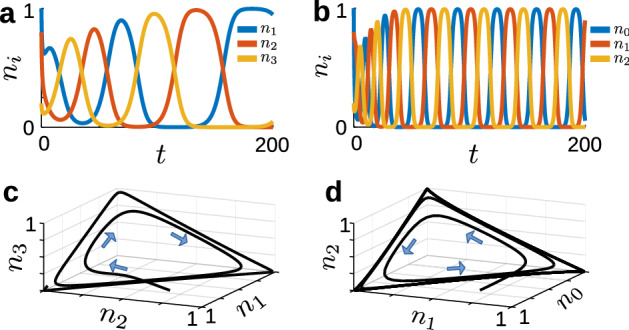
Comparison of deterministic minimal and three-pool models. **a** Relative population size $n_i$ as a function of *t* generated from Eq. (4) with $r=1$, $\alpha =0.8$, $\beta =1.3$, and $n(0)=(1,0.8,0.2)$. **b** Active fraction of neural pool $a_i$ as a function of *t* from Eq. (5) with $\tau =1$, $\gamma =2.4$, and $\mu =10^{-5}$ with same initial condition as **a**. **c** Solution from **a** plotted in phase space, with arrows indicating direction of motion. **d** Solution from **b** plotted in phase space, with arrows indicating direction of motion

To better understand the dynamics of the three-pool model, compared to the more traditional translations of heteroclinic cycling, we simulated the deterministic Eqs. (4) and (5) (see Fig. ). Fig. 1a shows that the minimal model exhibits the same heteroclinic cycling as May and Leonard’s original system. As expected, solutions converge to a two-dimensional surface in the vicinity of the plane $n_1+n_2+n_3=1$ in phase space (Fig. 1c). In contrast, in the three-pool model, when $\mu =0$ we recover a rescaled version of the original May–Leonard system. However, when $\mu >0$, the three-pool model does not exhibit heteroclinic cycling; instead, as Fig. 1b shows, it undergoes periodic oscillations. In this case, trajectories still converge to a two-dimensional surface in the vicinity of the triangular unit plane $n_1+n_2+n_3=1$, but with slightly different geometry as a result of the non-homogeneous forcing (see Fig. 1d).

In the following sections we introduce stochastic models corresponding to each of the three mean-field models discussed above. In order to constrain our choice of stochastic model, in each case we restrict consideration to models that obey mass-action kinetics. This choice allows us to leverage results from birth-death processes and complex-balanced equilibrium theory in order to study the long-time asymptotic behavior of each model. As a consequence of this modeling choice, the noise in our models will come from demographic stochasticity rather than, for example, scaled Gaussian noise typical of Langevin-type population models. By focusing on discrete-state population models, we aim to hew closely to the spirit of May and Leonard’s original work.

## Stationary distribution of general variance and minimal models

With our three mean-field models established, we now proceed with formulating stochastic implementations of each model, starting with the GV and minimal models from Eqs. (3) and (4). While, as we have discussed, there are many different stochastic systems that will all agree with the ODE systems in the mean-field limit, we will present what we consider the most natural interpretation of the underlying dynamics for discrete population sizes. This interpretation will take the form of discrete-state, continuous-time Markovian birth-death processes, so that the population vector ${\textbf{N}}=(N_1,N_2,N_3)$ is restricted to the lattice ${\mathbb {Z}}_{\ge 0}^3$. In general, we require a spontaneous birth process, a spontaneous death process, as well as death processes mediated by interspecies competition (“heterocidal” interactions) and within-species competition (“homocidal” interactions). In what follows, we will specify the strength of these interactions for each model, which defines the connectivity of the discrete state space and the average rate at which transitions between the different states occur.

### General variance model: total extinction

Following Higham (2008), Anderson and Kurtz (2015), and Wilkinson (2018), we adopt the formalism of stochastic mass-action kinetics and construct the reaction net for Eq. (3). Let $A_i$, $i\in \{1,2,3\}$, represent an individual member of species *i*, so that $N_i(t)$ is number of individuals $A_i$ at time *t*. Then, the mass-action kinetic system given by Eq. (3) is:

6 $$\begin{aligned} A_i&\xrightarrow {c_1}2A_i{} & {} (c_1 = b),{} & {} \text {birth}\end{aligned}$$

7 $$\begin{aligned} A_i&\xrightarrow {c_2}\emptyset{} & {} (c_2 = d),{} & {} \text {death}\end{aligned}$$

8 $$\begin{aligned} 2A_i&\xrightarrow {c_3}A_i{} & {} \left( c_3 = \frac{2}{\Omega }\right) ,{} & {} \text {homocidal competition} \end{aligned}$$

9 $$\begin{aligned} A_i+A_{i+1}&\xrightarrow {c_4}A_{i+1}{} & {} \left( c_4 = \frac{\alpha }{\Omega }\right) ,{} & {} \text {heterocidal competition} \end{aligned}$$

10 $$\begin{aligned} A_{i}+A_{i+2}&\xrightarrow {c_5}A_{i+2}{} & {} \left( c_5 = \frac{\beta }{\Omega }\right) .{} & {} \text {heterocidal competition} \end{aligned}$$

For readers unfamiliar with the stochastic reaction net formalism, we provide a brief review in Appendix A. Please note that in each of Eqs. (6–10) we take indicial addition of *i* cyclically. For each reaction, $c_j$ is the microscopic rate constant determining the propensity of the given reaction and is derived from the rate constants of the extensive variable ODE system (Higham 2008).

**Fig. 2:**
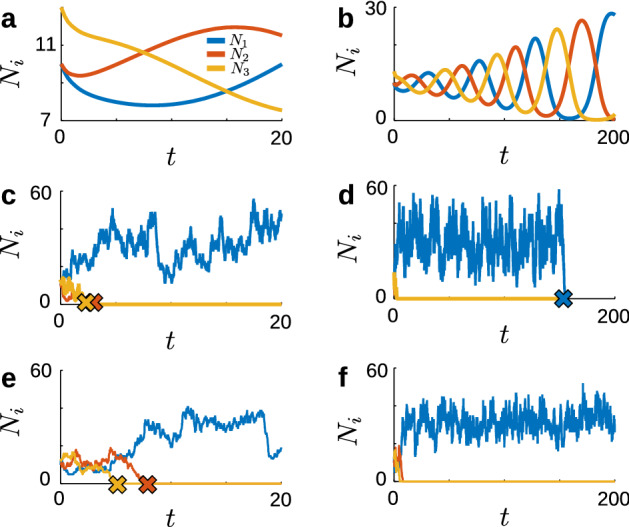
GV and minimal models of stochastic heteroclinic cycling. **a** Mean-field behavior of GV/minimal model from Eq. (3) with $\alpha = 0.8$, $\beta = 1.3$, $r=1$, $\Omega =30$, and ${\textbf{N}}(0)=\Omega \left( \frac{1}{3},\frac{1}{3},\frac{13}{30}\right)$. **b** Same as **a**, plotted for $0\le t \le 200$. **c** Stochastic realization of GV model from reaction system in Eqs. (6–10) with same parameters as **a**. Strikes (**x**) mark extinction events of each species. **d** Same as **b**, plotted for $0\le t \le 200$. Note that all species have gone extinct by the end of the simulation. **e** Stochastic realization of minimal model from reaction system in Eqs. (11–14) with same parameters as **a**. **f** Same as **e**, plotted for $0\le t \le 200$. In the minimal model, the last species survives in perpetuity

As stated above, we incorporate noise in the May–Leonard system by casting the model as a discrete-state Markov process with birth and death reactions specified by Eqs. (6–10). This formulation guarantees the noise in our stochastic model comes from demographic stochasticity, arising from the inherent randomness in transitions between discrete states. Adding other types of noise, such as Gaussian white noise, to some deterministic systems can lead to “stochastic limit cycling” (Pérez-Cervera et al. 2021) with finite mean period (Armbruster et al. 2003; Bakhtin 2011; Shaw 2014; Thomas and Lindner 2014; Cao et al. 2020), creating a strong central peak in the power spectrum (Giner-Baldó et al. 2017). Such systems typically exhibit a crater-like distribution that is nevertheless a stationary distribution, albeit with nonzero circulation. In contrast, for the GV model, we find that while the inclusion of discrete population noise from demographic stochasticity does prevent the trapping behavior that leads to heteroclinic cycling in deterministic ODE models, it does not lead to long-term “stochastic limit cycle”-like behavior with finite mean period. Rather, as Fig.  shows, trajectories exhibit several cycles around the fixed point of the deterministic system before colliding with boundaries, resulting in population extinctions. Figure 2b,d shows a sample trajectory of this system generated via Gillespie’s stochastic simulation algorithm (Gillespie 1977; Higham 2008). While short-term dynamics of the mean-field model Eq. (3) evolve slowly from initial conditions (Fig. 2a,b), the stochastic GV system quickly exhibits extinction of two of the three species (Fig. 2c). This result is consistent with May and Leonard’s prediction: while intensive variables can become arbitrarily close to zero, extensive variables taking discrete values will eventually drop to exactly zero. However, rather than leading the third “winning" species to dominate in perpetuity, on a longer time scale (Fig. 2d) the winning population also ultimately suffers a downward fluctuation leading to its own extinction. Indeed, the following proposition establishes that the unique stationary distribution for the general variance stochastic model is total extinction.

#### Proposition 1

Let ${\textbf{N}}=(N_1,N_2,N_3)$ be the vector of individuals in each population of the GV model. If the per capita death rate $d>0$, then the unique stationary distribution of the reaction system Eqs. (6–10) is $\pi ({\textbf{N}})=\delta ({\textbf{N}})$.

We provide a proof in Appendix B.

Proposition 1 tells us that the GV model will exhibit total extinction in the long-time limit, independent of initial conditions, with probability one. This result recalls that of Vellela and Qian (2007), in which the authors demonstrated that for the single-population Keizerator reaction system, the mean-field system converges to a nontrivial equilibrium while the stochastic system converges to total extinction (albeit with mean extinction times that are exponentially long in the system size). We can explain this behavior by the fact that our reaction system includes individual birth ($N_i\rightarrow 2N_i$) and death ($N_i\rightarrow \emptyset$) reactions (Allen 2010). As we will see in Sect. 3.2, removing the death reaction fundamentally changes the stationary behavior of the model.

### Minimal model: persistence of a single species

We previously noted that Eq. (4) is a special case of Eq. (3), obtained by setting $d=0$ and $b=r$. Setting $d=0$ in the ODE system is equivalent to removing the individual death reactions $A_i\rightarrow \emptyset$, which changes the connectivity of the underlying state space and, as we will show, fundamentally changes the long time behavior of the stochastic system. With this change, the reaction system takes the form (for $i\in \{1,2,3\}$, as before):

11 $$\begin{aligned} A_i&\xrightarrow {c_1}2A_i{} & {} (c_1 = r),{} & {} \text {birth} \end{aligned}$$

12 $$\begin{aligned} 2A_i&\xrightarrow {c_2}A_i{} & {} \left( c_2 = \frac{2}{\Omega }\right) ,{} & {} \text {homocidal competition}\end{aligned}$$

13 $$\begin{aligned} A_i+A_{i+1}&\xrightarrow {c_3}A_{i+1}{} & {} \left( c_3 = \frac{\alpha }{\Omega }\right) ,{} & {} \text {heterocidal competition} \end{aligned}$$

14 $$\begin{aligned} A_i+A_{i+2}&\xrightarrow {c_4}A_{i+2}{} & {} \left( c_4 = \frac{\beta }{\Omega }\right){} & {} \text {heterocidal competition}. \end{aligned}$$

Fig. 2c, d, e, and f contrast Gillespie simulations of the GV model and the minimal model. In both cases two population extinctions occur quickly (Fig. 2c, d), but in the minimal model the third population does not go extinct (Fig. 2f). In the minimal model, once the system reduces to a single species, the only death mechanism is homocidal competition, which requires at least two individuals. Therefore the total extinction state is not accessible from non-trivial initial conditions, which guarantees a distinct stationary distribution from the GV model. The framework of complex-balanced equilibria from Horn and Jackson (1972) and Anderson and Kurtz (2015) allows us to obtain this stationary distribution for the system, as given in Proposition 2:

#### Proposition 2

Let ${\textbf{N}}=(N_1,N_2,N_3)$ be the population vector of the minimal model Eqs. (11–14). The reaction system Eqs. (11–14) has four distinct stationary distributions. Three may be expressed as component-wise stationary distributions of the form

15 $$\begin{aligned} \pi (N_i)=\frac{(r\Omega )^{N_i}}{N_i!(e^{r\Omega }-1)}\delta (N_{i+1})\delta (N_{i+2}), \end{aligned}$$

for $i\in \{1,2,3\}$, $N_i\ge 1$, with $\delta (x)$ being the distribution with unit probability at $x=0$, and with index addition taken cyclically on $\{1,2,3\}$. The fourth is $\pi ({\textbf{N}})=\delta ({\textbf{N}})$.

We provide a proof in Appendix C. Figure  shows a comparison of the analytic stationary distribution from Eq. (15) with the empirical distribution from Gillespie simulations. We can see that the two results show excellent agreement, even when the system size $\Omega$ takes on non-integer values.

**Fig. 3:**
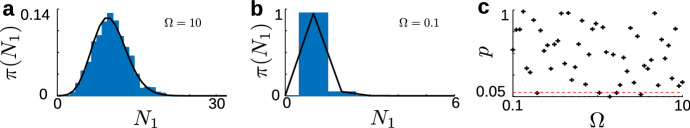
Comparison of analytical and empirical stationary distributions for the minimal variance model. **a** Blue bars show histogram of empirical stationary distribution from Gillespie simulations with $\alpha =0.8$, $\beta =1.3$, $r=1$, and $\Omega =10$. Black curve shows stationary distribution calculated from Eq. (15) with $\Omega =10$. **b** Same as **a**, with $\Omega =0.1$. **c** *p*-values from $\chi ^2$ goodness-of-fit test between empirical stationary distribution from Gillespie simulations and analytical stationary distribution from Eq. (15) with varied $\Omega$. Dashed line shows standard significance level $\alpha =0.05$; *p*-values above the dashed line indicate good agreement between the distributions (color figure online)

Comparing Propositions 1 and 2, the stationary behaviors of the GV and minimal models are in fact distinct. While two of the populations will go extinct in both models, the GV model converges to total extinction while the minimal model converges to a truncated Poisson distribution representing stochastic logistic growth. Although the total extinction state is a stationary distribution for the minimal model, it is not accessible from nontrivial initial conditions. This comparison illustrates the well-known fact that two stochastic models both consistent with the same mean-field deterministic model can have fundamentally different long-term behavior.

## Transient behavior of the minimal model

While we have thus far restricted our investigation to long-time asymptotic behaviors, we may also study the dynamics of extinction over intermediate times. The order and timing of extinctions is important in conservation ecology, where it is crucial to determine if and when intervention is required to prevent population collapse (Shaffer 1981; Purvis et al. 2000). Gillespie simulations suggest that the cycle length of the stochastic May–Leonard system, conditioned on non-extinction, has finite mean (Fig. 2). Taking this observation together with the stationary distribution results from Sect. 3, we can reasonably expect to estimate both the ordering of extinction events and their times of the stochastic system. Because both the GV and minimal models exhibit similar transient behavior, we will restrict our investigations to the minimal model, as the results will be more clear due to its lower variance.

### Distribution and ordering of extinction events

**Fig. 4:**
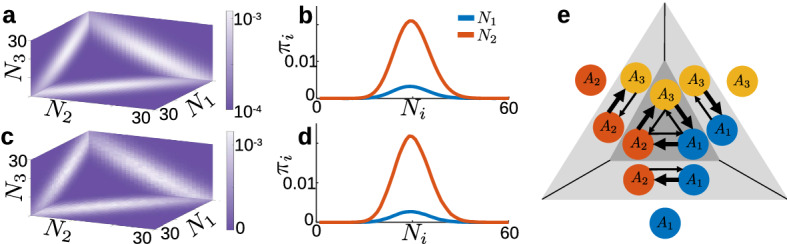
Ordering of extinction events in the minimal-variance model. **a** Exact distribution for first extinction event obtained by solving the discrete first-passage location problem ($r=1$, $\alpha =0.8$, $\beta = 1.3$, $\Omega =30$, $N(0)=\frac{\Omega }{3}(1,1,1)$). For an unbiased initial condition, the distribution exhibits three-fold rotational symmetry, which implies all three populations are equally likely to go extinct. **b** Exact distribution for second extinction event, conditioned on $A_3$ going extinct first, obtained by solving the discrete first-passage location problem. Red (upper) curve: Density of population $A_1$ when population $A_2$ goes extinct. Blue (lower) curve: Density of population $A_2$ when population $A_1$ goes extinct. The red (upper) density has more mass than the blue (lower) density, which implies that once $A_3$ goes extinct, $A_2$ is more likely to go extinct next. See text for additional details. **c** Empirical distributions for first extinction event, obtained from $10^6$ Gillespie simulations with the same parameters as in **a**. **d** Empirical distributions for second extinction event, conditioned on $A_3$ going extinct first, obtained from Gillespie simulations with $10^6$ initial states sampled from the plane $N_3=0$ in **c**. **e:** Schematic showing competition interactions and ordering of extinction events. Thicker arrows indicate stronger competitive interactions (color figure online).

To study the ordering of extinction events in the minimal model, we found the distribution of hitting locations on the coordinate planes $N_i=0$ for $i\in \{1,2,3\}$ from a fixed initial condition ${\textbf{N}}(0)=\frac{\Omega }{3}(1,1,1)$. This distribution gives the relative probability of extinction of each species from this starting condition. In addition, it gives us the conditional density of, for example, species 2 and 3, conditioned on species 1 going extinct first. We formulated the first-passage location problem as

16 $$\begin{aligned} {\mathcal {L}}\pi ={\textbf{e}}_s, \end{aligned}$$

where ${\mathcal {L}}$ is the infinitesimal generator matrix associated with the discrete master equation, *s* is a fixed absorbing state, $\pi$ is the probability of hitting *s* as a function of initial condition, and ${\textbf{e}}_s$ is the standard basis vector. We imposed absorbing boundary conditions along the coordinate planes $N_i=0$ and adjoint reflecting boundary conditions along the planes $N_i=2\Omega$ to ensure a well-posed numerical problem in which probability conservation is guaranteed. For more details about this construction, see Appendix D. Fig. a shows the solution of this linear system; we can see that for an initial condition along the vector (1, 1, 1), the distribution of absorption locations exhibits a three-fold rotational symmetry about the initial condition. The majority of the distribution is located near the intersections of the plane $N_1+N_2+N_3=\Omega$ with the three absorbing coordinate planes. These results suggest that, for a symmetrically-distributed initial condition, all three populations are equally likely to go extinct. To confirm these findings, we also found the first-hitting distribution empirically, shown in Fig. 4c, using Gillespie simulations. We can see that the two approaches show good agreement over the entire domain.

Assuming WLOG that $A_3$ is the first population to go extinct, we formulated the first-hitting problem for the two-dimensional subsystem to find the absorption distribution of the remaining two species conditioned on extinction of $A_3$. Using the same approach from the full three-dimensional system, with initial conditions weighted by the distribution in Fig. 4a over the plane $N_3=0$, we obtained the distributions shown in Fig. 4b. The upper (red) curve labeled “$N_2$” shows the density of $N_1$ at the time $A_2$ goes extinct. Similarly, the lower (blue) curve labeled “$N_1$” shows the density of $N_2$ at the time $A_1$ goes extinct. The area under each curve gives the conditional probability that the corresponding population goes extinct, given that $A_3$ goes extinct first; note that the summed area under the two curves equals unity. From these results, we can see that once $A_3$ goes extinct, $A_2$ is much more likely to go extinct than $A_1$. We again confirmed our these results using Gillespie simulations (Fig. 4d) and found good agreement ($\chi ^2$ goodness-of-fit test, $p=0.96>0.05$).

Combining these two results, we can see that there is a distinct pattern to the extinctions in the minimal model, which is schematized in Fig. 4e. In the full three-dimensional system, the likelihood of each extinction is determined by the initial conditions; any initial condition along the vector (1, 1, 1) results in an equal probability of first extinction. Once one population goes extinct, a second population quickly goes extinct because of the imbalance in competition rates $\alpha$ and $\beta$, leaving a sole surviving species. For example, if species 3 goes extinct first, then it is more likely that species 2 goes extinct next, leaving species 1 to dominate over long times. This pattern is reminiscent of the age-old saying “the enemy of my enemy is my friend,” as species 1, which is out-competed by species 3, survives because species 2 out-competes species 3.

### Extinction times in the minimal model

**Fig. 5:**
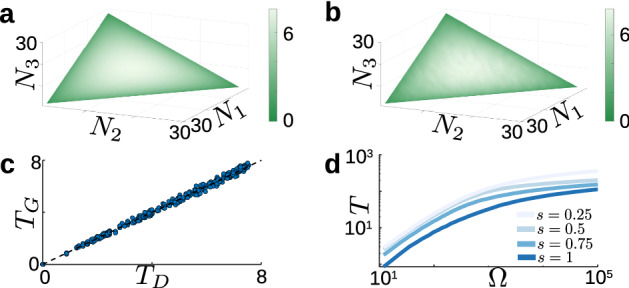
Timing of first extinction in the minimal model. **a** Exact mean time to first extinction event, obtained by solving the discrete first-passage time problem ($r = 1$, $\alpha =0.8$, $\beta = 1.3$, $\Omega =30$), from initial conditions in the plane $\Pi =\{N_1+N_2+N_3=\Omega /3\}$. **b** Empirical expected time to first extinction event, calculated using $10^4$ Gillespie simulations over $\Pi$ with the same parameters as in **a**. **c** Scatter plot of mean extinction times at every initial condition. Abscissa: Exact time $T_D$, from discrete backward equation. Ordinate: Empirical mean time $T_G$, from $10^4$ samples. **d** Empirical mean time to first extinction event, obtained from $10^4$ Gillespie simulations, as a function of $\Omega$. Several values of *s* along the segment $\left[ \Omega (1-s)+\frac{\Omega s}{3},\frac{\Omega s}{3},\frac{\Omega s}{3}\right]$ are superimposed

In order to find the exact mean time to first extinction, we construct the first-passage time problem

17 $$\begin{aligned} {\mathcal {L}}\varvec{\tau }=\mathbf {-1}, \end{aligned}$$

where ${\mathcal {L}}$ is the same infinitesimal generator matrix from Eq. (16), $\tau$ is the vector of mean absorption times as a function of initial condition, and $\mathbf {-1}$ is a vector of −1’s. We impose absorbing boundary conditions on the coordinate planes $N_i=0$, and adjoint reflecting boundary conditions on the planes $N_i=2\Omega$, as in the first-passage location problem (Sect. 4.1). Using this approach, we obtained the mean first-extinction time for all initial states in the domain. For ease of visualization, Fig. A and B show $\tau$ restricted to the plane $\Pi =\{N_1+N_2+N_3=\frac{\Omega }{3}\}$. (We note that trajectories with initial conditions away from $\Pi$ quickly approach a small neighborhood of this plane, so mean extinction times on the plane are representative of mean extinction times from most starting locations in the interior of the domain.) Fig. 5a shows a slice of the mean first-extinction time along the plane $\Pi$. As expected, the extinction times as a function of initial condition have three-fold rotational symmetry. Moreover, the time is largely determined by the distance between the initial condition and the deterministic fixed point $\frac{\Omega }{3}(1,1,1)$.

To confirm the results from the discrete first-passage time problem, we also used large-sample Gillespie simulations with initial condition taken over $\Pi$. Fig. 5b shows the empirical mean first-extinction time as a function of starting location. Comparing the exact and approximate results, we found good agreement (*t*-test, averaged over initial conditions: $\langle p({\textbf{N}})\rangle _{\Pi }\approx 0.51>0.05$). To illustrate this agreement further, in Fig. 5c we plot each initial condition in a scatter plot: the abscissa is the exact mean extinction time found using the discrete backward equations ($T_D$) and the ordinate is the empirical mean extinction time found using Gillespie simulations ($T_G$). The two methods show excellent agreement for initial states near the coordinate planes (when $T_D$ is small) and have a slightly increased variance when the initial state is close to the deterministic fixed-point (when $T_D$ is large). Nevertheless $T_D$ and $T_G$ show excellent agreement over the entirety of $\Pi$. This result demonstrates that large-sample Gillespie simulations give a good approximation of the exact mean extinction times, and justifies the use of Gillespie simulations for large-$\Omega$ systems where the exact solution becomes intractable (e.g. $\Omega \gtrsim 60$).

In order to study the effect of system size on mean extinction time for $\Omega >30$, we relied on Gillespie simulations. From our previous simulations for a fixed $\Omega =30$ system, we observed that time to first extinction has three-fold rotational symmetry and largely depends on the distance from the deterministic fixed point. Therefore we considered initial conditions along the segment connecting $(\Omega ,0,0)$ and $\frac{\Omega }{3}(1,1,1)$, where we parameterized the distance along this segment using the parameter $s\in [0,1]$. Using this parameterization, we varied $\Omega$ and *s* and estimated the time to first extinction, shown in Fig. 5d. As *s* increases and the initial condition moves closer to the deterministic fixed point, the mean extinction time increases across all values of $\Omega$; this trend is consistent with the behavior we observe in the small-$\Omega$ system.

## Three-pool model: stochastic oscillations

In the previous sections, we showed that modifying a single reaction in the stochastic model (removing the individual death reaction) led to distinct asymptotic dynamics. However, both the GV and minimal models share the same mean-field behavior, and both produce transient dynamics that may be described as noisy heteroclinic cycling. In contrast, the three-pool model for a neuromotor central pattern generator (CPG) in Eq. (5) has a non-homogeneous term, $\mu$, that steers trajectories away from the fixed points in the corners of the boundaries, preventing heteroclinic cycling, as demonstrated in Fig. 1b, d. Recall that in Eq. (5), each extensive variable $N_i$, $i\in \{0,1,2\}$, represents the integer number of active neurons in the *i*-th pool, ranging from $N_i=0$ (inactive) to $N_i=\Omega$ (fully active). The non-homogeneous forcing term of the CPG model, controlled by the parameter $\mu$, can be interpreted as endogenous activation of each pool of motor neurons. When $\mu >0$ the resulting deterministic system exhibits finite-period oscillations, converting heteroclinic cycling into finite-period limit cycle behavior (see Fig. 1), with prolonged dwell times near the saddle points and a period that can be sensitively controlled by the endogenous activation parameter.

To develop a stochastic implementation of the three-pool model, we use a birth-death process formalism as in Sect. 3. Letting $A_i$ represent an active cell in the *i*-th pool and $I_i$ represent an inactive cell in the *i*-th pool, the three-pool reaction net is:

18 $$\begin{aligned} A_i+I_i&\xrightarrow {c_1}2A_i{} & {} \left( c_1 = \frac{1}{\tau \Omega }\right) ,{} & {} \text {self-activation} \end{aligned}$$

19 $$\begin{aligned} A_i+A_{i+1}&\xrightarrow {c_2}I_i+A_{i+1}{} & {} \left( c_2 = \frac{\gamma }{\tau \Omega }\right) ,{} & {} \text {inhibition of }i\text { by }i+1\end{aligned}$$

20 $$\begin{aligned} I_i&\xrightarrow {c_3}A_i{} & {} \left( c_3 = \frac{\mu }{\tau }\right) ,{} & {} \text {endogenous activation} \end{aligned}$$

where indicial addition over *i* is taken cyclically. Note that the *per capita reaction rates* in Eqs. (18–20) inversely depend on the system size $\Omega$. This relationship means that increasing $\Omega$ keeps the *net mean reaction rates* constant. Additionally, the endogenous activation $\mu$ enters into the reaction $I_i\rightarrow A_i$. Because the total population of cells in each pool remains fixed over time, this reaction ensures that even if one population becomes fully inactive, it will eventually become active again, after some delay. Whereas population extinction is a feature of the previous two models, in the three-pool model the neural activity can never be permanently extinguished. Consequently the neural activity oscillation persists indefinitely, albeit with a randomly varying cycle length.

Both endogenous activation and noise intensity have been suggested as potential mechanisms for regulating the frequency of cycling in CPG models built on a dynamical architecture of heteroclinic cycling (Shaw 2014; Shaw et al. 2012, 2015; Lyttle et al. 2017). To calculate the “cycle length” of a stochastic trajectory, we define three Poincaré sections $P_i$, $i\in \{1,2,3\}$, each of which is a triangle with verticies (0, 0, 0), $(\Omega ,\Omega ,\Omega )$, and $\Omega {\textbf{e}}_i$, where ${\textbf{e}}_i$ is the *i*th canonical basis vector for ${\mathbb {R}}^3$. We start each trajectory at $(\Omega ,0,0)\in P_1$ and calculate the times for the trajectory to travel from $P_1$ to $P_2$, $P_2$ to $P_3$, and $P_3$ to $P_1$, thereby completing a full circuit orbiting the central diagonal of the domain. We define the sum of these three times as the cycle length. The three-pool model specified in Eqs. (18–20) allows us to investigate the relative contributions of both activation (controlled by $\mu$) and noise (controlled by the system size $\Omega$) to regulating the mean oscillation period of the CPG model.

**Fig. 6:**
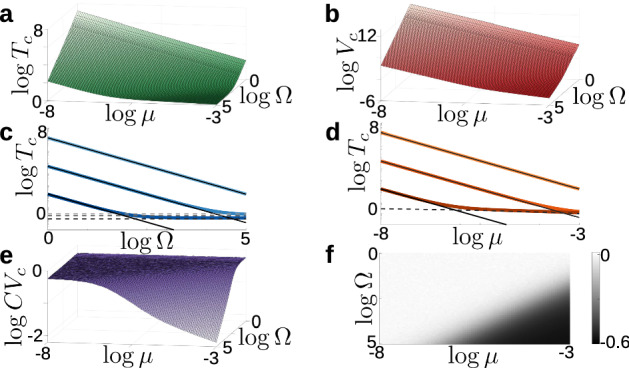
Cycle length statistics for the three-pool model. **a** Mean cycle length $T_c$ as a function of system size $\Omega$ and excitation parameter $\mu$. Mean calculated at each parameter set using $10^4$ samples from Gillespie simulations. **b** Variance in cycle length as a function of $\Omega$ and $\mu$. **c** Mean cycle length as a function of $\Omega$, with several values of $\mu$ superimposed. Solid thin lines show the approximate mean cycle length given by Eq. (21), and dashed lines show cycle length of deterministic system from Eq. (5) for the given value of $\mu$. **d** Mean cycle length as a function of $\mu$, with several values of $\Omega$ superimposed. Solid thin lines show approximate cycle length given by Eq. (21), and dashed line shows deterministic cycle length as a function of $\mu$. **e** Coefficient of variation (CV) of the cycle length as a function of $\Omega$ and $\mu$. **f** Difference in CV from Gillespie samples $(CV_c)$ and CV from Gamma distribution approximation $(CV_a)$, calculated as $\Delta CV=CV_c-CV_a$, as a function of $\Omega$ and $\mu$

Figure  illustrates how the population size $\Omega$ and activation strength $\mu$ influence the cycle length. In order to cover a wide range of system sizes, we utilized Gillespie simulations. Figure 6a shows the empirical mean cycle length for varied $\Omega$ and $\mu$; we observe that larger parameter values cause faster oscillations, on average. Additionally, as $\Omega$ increases, the mean period approaches a value that depends solely on $\mu$; this value is the deterministic period from the mean-field equations in Eq. (5). We calculated the empirical variance of the cycle length, shown in Fig. 6b, and found that the variance also decreases when either $\Omega$ or $\mu$ are increased. These results suggest that both $\mu$ and $\Omega$ could contribute to controlling the frequency of neural activity. For example, consider a relatively slow system, with small $\Omega$ and small $\mu$. This system can be sped up by either increasing $\mu$, which increases endogenous activation noise and drives activity further away from the saddle points, or by increasing $\Omega$, which moves the system closer to the mean-field limit, thereby decreasing noisy fluctuations caused by demographic stochasticity. Additionally, both parameters have similar influence on the variance of the cycle length. Recent work has shown that the feeding CPG of the marine mollusk *Aplysia californica* recruits additional motor neurons when the organism encounters unexpected resistance in swallowing food (Gill and Chiel 2020; Gill 2020), and that variability of motor neuronal activity is reduced for those components of feeding behavior that matter most for task fitness (Cullins et al. 2015b). Although the isolated three pool model considered here lacks important circuit components (such as sensory feedback (Cullins et al. 2015a)), the relative sensitivity of the cycle time variance to $\mu$ versus $\Omega$ could nevertheless suggest experimentally testable questions. For instance, one could probe experimentally whether the variability in the motor pattern decreases or increases when subjected to larger external loads.

Figure 6a, b exhibit a large region of parameter space in which the mean and variance both vary linearly on a log scale with both $\Omega$ and $\mu$. To explain this observation, we developed an approximate expression for the average period as a function of $\Omega$ and $\mu$. Consider the discrete system, which forms a cubic lattice, and suppose the population vector is currently $(\Omega ,0,0)$. While there are three possible transitions away from this state, the only transition that pushes the system forward along a cycle is the transition $(\Omega ,0,0) \rightarrow (\Omega ,1,0)$. The time of this transition is exponentially distributed with rate parameter $\frac{\Omega \mu }{\tau }$. Once this transition occurs, the subsequent transitions are more rapid, and push the system to the corner $(0,\Omega ,0)$, where the process repeats itself. Because of the differing timescales of these transitions, we can approximate the cycle dynamics as a sequence of three “rate-limiting steps”, each with transition times that are iid exponentially distributed with parameter $\frac{\Omega \mu ^{}}{\tau }$. The sum of these three times follows a Gamma distribution with parameters $(\alpha ,\beta )=\left( 3,\frac{\tau }{\mu \Omega }\right)$. This distribution predicts the mean cycle length and coefficient of variation to be

21 $$\begin{aligned} T_a\approx \frac{3\tau }{\Omega \mu },\quad \quad \text {CV}_a\approx \frac{1}{\sqrt{3}}. \end{aligned}$$

To verify the Gamma distribution approximation and the predicted mean cycle length given by Eq. (21), we plot the average period as a function of $\Omega$ for fixed $\mu$, and as a function of $\mu$ for fixed $\Omega$ (solid thin lines) against the empirical average period (thick colored lines) in Fig. 6c, d, respectively. The Gamma distribution and Gillespie simulation results agree in the region of small $\Omega$ and small $\mu$; however, the Gamma distribution approximation breaks down as $\Omega$ increases and the number of different transition paths between fixed points increases. To further validate the Gamma distribution approximation, in Fig. 6e we plot the coefficient of variation (CV) of the empirical cycle length and found that the CV is approximately constant for a large region of parameter space. Comparing our empirical CV to the predicted value Eq. (21) in Fig. 6f, we found that this heuristic interpretation holds for a large range of parameters.

## Discussion

In this work, we studied both transient and long-term behavior in several stochastic versions of May and Leonard’s heteroclinic cycling model, introducing noise via demographic stochasticity under a variety of assumptions. Although two of our models (the general-variance (GV) and minimal models) coincide with the classical May–Leonard system in Eq. (1) in the mean-field limit, we found that these stochastic versions are guaranteed to undergo population extinctions in finite time. Moreover, by eliminating the individual death reactions in the GV model to obtain the minimal model, we proved that the stationary distribution changes from total extinction of all species (in the GV model) to extinction to a sole survivor that follows a truncated Poisson distribution (in the minimal model). We also studied a variant of the model representing a three-pool neural system. In this version, we added a single reaction to introduce endogenous excitation of each neural population; in an ecological context a similar modification can be thought of as representing immigration. This additional reaction yielded a system that not only avoids permanent extinctions, but has a finite mean cycle time that depends on the size of each neural pool ($\Omega$) and the strength of endogenous excitation ($\mu$). Using an intuitive rate-limiting step argument, we found an approximation to the mean cycle length that showed good agreement in both mean and variance with Gillespie simulations. As elements of a potential control scheme for a neural central pattern generator, it is worth noting that although both $\Omega$ and $\mu$ provide potential control parameters, their effects on the mean and variance of the cycle time are similar for a wide range of parameters, meaning that the mean and variance cannot be controlled independently of one another.

Throughout our investigation, we limited attention to stochastic models with mass-action kinetics. This focus allowed us to formulate our models as multi-dimensional birth-death processes and leverage results from complex balanced equilibrium theory to find the stationary distribution of the GV and minimal models. While others, such as Reichenbach et al. (2006) and Yahalom et al. (2019), have studied cyclic stochastic population models, their results required taking continuum limits of the state space and linearizing the resulting dynamics about fixed-points of the associated deterministic system. As a result, their models employed Gaussian white noise rather than discrete population noise, which can lead to inconsistent treatment of small population dynamics leading to extinction (Strang et al. 2019). Our approach avoided these potential difficulties and guaranteed that demographic stochasticity was the only source of noise in our models. Discrete-state continuous time models of neuronal populations, based directly on the Wilson-Cowan formalism, has been used to investigate how noise arising from finite neural populations can induce exotic dynamics such as avalanches (Benayoun et al. 2010), limit cycles, and quasicycles (Wallace et al. 2011). To the best of our knowledge, the effects of finite population noise on neural models with an underlying *heteroclinic* cycle have not previously been examined.

Determining stationary distributions for multiple interacting species remains a challenging task. Even for one-dimensional birth-death processes, authors such as Allen (2010) have shown that extinction is not guaranteed if the chain is simply connected to an absorbing state, when the state space is unbounded. Additionally, projecting a multi-dimensional Markov processes into a single dimension often results in a non-Markovian process. Thus, our results required careful application of first-principles Markov chain theory to multi-dimensional systems to obtain stationary distributions. While we formulated our system using a discrete state space, work in the field of stochastic permanence has determined stability and extinction conditions for continuous-state stochastic systems (Hofbauer et al. 1979, 1998; Jansen and Sigmund 1998). Benaïm et al. (2008) and Schreiber et al. (2011) developed notions of stability for systems that are stochastically perturbed in time, which can be interpreted as environmental fluctuations that impact fitness functions of different species. Recent work by Hening et al. has further extended this work to stochastic switching systems that can exhibit discontinuous changes in conditionally deterministic dynamics. Such hybrid systems occur in neuroscience (Anderson et al. 2015b), cell biology (Bressloff 2014), and ecology (Hening and Li 2021; Hening et al. 2021). In an ecological context, Hening et al. (2020) classified the resulting systems by analyzing their stationary distributions. Our models are not influenced by external stochasticity that comes from environmental fluctuations and thus do not benefit from the analytical tools of stochastic permanence theory. Future work extending our analysis to more naturalistic, dynamic environments would likely require the use of such tools developed by these authors.

To verify our analytic results, we ran both large-scale Gillespie simulations and constructed first-passage problems as sparse linear systems. While these two numerical approaches showed good agreement, we could only leverage the exact results from the master equation for small system sizes. This limitation is a consequence of our choice to use a birth-death formalism for all the stochastic models: the infinitesimal generator matrix ${\mathcal {L}}$ that is required for solving first-passage problems has ${\mathcal {O}}\left( \Omega ^3\right)$ scaling, and we quickly reached hardware limitations when trying to vary $\Omega$ over several orders of magnitude. Future work using the discrete system may require approximating the operator to make the first passage problem tractable. Safta et al. (2015) have developed a hybrid discrete-continuum approximation of the forward operator, the adjoint of the infinitesimal generator matrix, that involves partitioning the state space, taking a continuum limit within each partition, and simulating a continuous flow within partitions and discrete transitions between partitions. In future work, this approach could be extended to create a hybrid approximation to the backward operator ${\mathcal {L}}$ to solve first-passage time problems, which would allow us to obtain the extinction time statistics for a larger range of system sizes semi-analytically.

## Figures and Tables

**Fig. 7:**
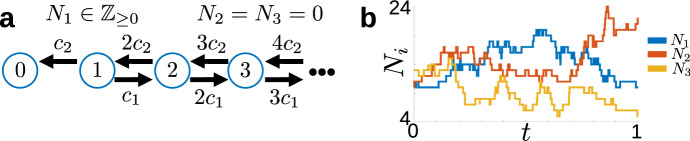
Structure of stochastic models. **a** Topology of the discrete state space of the GV model for the case $N_1\in {\mathbb {Z}}_{\ge 0}$ and $N_2=N_3=0$. Arrows are labeled with the intensity of the Poisson process corresponding to the transitions between the given states, calculated using the propensity functions $\lambda _k$. **b** Sample path of the GV system from Eq. (6–10) obtained using Gillespie’s algorithm. Time axis is enlarged to show piecewise-constant structure of trajectories. Parameters are the same as those from Fig. 2**c**

## Data Availability

See https://github.com/nwbarendregt/StochasticHC for the MATLAB code used to generate all results and figures.

## Appendix A: Details of stochastic mass-action kinetics

Here we give more details regarding the evolution of our stochastic systems under reaction nets as explained by Higham (2008), Anderson and Kurtz (2015), and Wilkinson (2018). For simplicity, we show here the details for the stochastic GV model given by Eqs. (6–10); the details for the minimal and three-pool models are similar. We start by assuming the population vector ${\textbf{N}}=(N_1,N_2,N_3)$ evolves according to a discrete-state, continuous-time Markov process with state space ${\mathbb {Z}}_{\ge 0}^3$. To describe the dynamics of ${\textbf{N}}$, we define the connectivity of the state space, as well as the probability of transitioning between connected states. We define the connectivity of the state space through the possible interaction terms, represented in the language of molecular reactions. For example, the reaction $A_1\xrightarrow {c_1}2A_1$, given by Eq. (6) with $i=1$, implies the states $(N_1, N_2, N_3)$ and $(N_1+1, N_2, N_3)$ are connected for all $N_1\in {\mathbb {Z}}_{>0}$. The resulting change in ${\textbf{N}}$ caused by each reaction can be represented by a stoichometric vector $\xi _k$, with the index *k* denoting the change in ${\textbf{N}}$ caused by reaction *k*. For this example reaction, the stoichometric vector is $\xi =(1,0,0)$.

To define the probability of transitioning between states, we follow Anderson and Kurtz (2015) and describe the dynamics of the state vector ${\textbf{N}}(t)$. and describe the dynamics of the state vector ${\textbf{N}}(t)$. Let $k\in \{1,\dots ,K\}$ index the set of all possible reactions, $\{\xi _k\}$ be the set of stoichometric vectors associated with each reaction, and $\{Y_k\}$ be a set of independent, unit Poisson processes. Then, under the standard interpretation of discrete stochastic chemical reaction kinetics, the state vector ${\textbf{N}}(t)$ evolves according to the stochastic equation

A1 $$\begin{aligned} {\textbf{N}}(t)={\textbf{N}}(0)+\sum _{k=1}^K\xi _kY_k\left( \int _0^t\lambda _k\left( {\textbf{N}}(s)\right) \,ds\right) , \end{aligned}$$

where $\lambda _k$ is the propensity, or hazard, function that specifies the intensity of the Poisson process $Y_k$. For reactions obeying mass-action kinetics, these propensity functions are given as follows:

A2 $$\begin{aligned} \emptyset&\xrightarrow {c_1}A_1{} & {} \lambda _1=c_1,{} & {} \text {zeroth-order} \end{aligned}$$

A3 $$\begin{aligned} A_1&\xrightarrow {c_2}A_2{} & {} \lambda _2=c_2N_1,{} & {} \text {first-order}\end{aligned}$$

A4 $$\begin{aligned} A_1+A_2&\xrightarrow {c_3}A_3{} & {} \lambda _3=c_3N_1N_2,{} & {} \text {multi-species second-order} \end{aligned}$$

A5 $$\begin{aligned} 2A_1&\xrightarrow {c_4}A_2{} & {} \lambda _4=c_4\frac{N_1(N_1-1)}{2}{} & {} \text {single-species second-order}. \end{aligned}$$

The propensity function for second-order reactions of a single species given by Eq. (A5) arises from a combinatorial argument; more details can be found in Higham (2008) and Wilkinson (2018). Note that the propensity functions only depend on the current state ${\textbf{N}}$ (i.e., they do not depend on the right-hand side of the reactions in Eqs. (A2–A5)). For this reason, every system considered is Markovian. In each of our stochastic models, every reaction belongs to one of these four classes, albeit with a variety of right-hand sides. Fig. a shows a simplified schematic of the state space topology and propensity functions for the GV model restricted to $N_2=N_3=0$.

To simulate realizations of the stochastic process given by Eq. (A1), we use Gillespie’s algorithm (Gillespie 1977; Higham 2008). At a time *t*, we first calculate the propensity functions $$\{\lambda _k\}$$ using the current state vector $${\textbf{N}}(t)$$. Next, we draw the time $$t+\delta t$$ of the next reaction given by $$\delta t\sim \text {exp}\left( \sum _{k=1}^K\lambda _k\right) $$, which is the minimum of the set of exponentially-distributed random variables with distributions $$\{\text {exp}(\lambda _k)\}$$. Finally, we determine the reaction that takes place by weighting each possible reaction *j* by the propensity ratio $$\frac{\lambda _k}{\sum _{j=1}^K\lambda _j}$$. Fig. 7b shows a zoomed-in sample path for the GV model (Eq. 6–10) obtained from this stochastic simulation algorithm. Because the dynamics of the system are represented with Poisson processes, all trajectories are piecewise constant. To be more precise, they are càdlàg processes: every piecewise-constant component *f*(*t*) is defined on an interval $$[t_1,t_2)$$, is continuous from the right (i.e., the limit $$f\left( t\rightarrow t_1^+\right) $$ exists and equals $$f(t_1)$$), and has limits from the left (i.e., the limit $$f\left( t\rightarrow t_2^-\right) $$ exists). For further details of càdlàg processes, see Anderson and Kurtz (2015).

## Appendix B: Proof of proposition 1

For the reader’s convenience, we restate Proposition 1:

*Let* ${\textbf{N}}=(N_1,N_2,N_3)$ *be the vector of individuals in each population of the GV model. If the per capita death rate* $d>0$, *then the unique stationary distribution of the reaction system Eqs.* (6–10) *is* $\pi ({\textbf{N}})=\delta ({\textbf{N}})$*.*

Our proof follows ideas similar to Vellela and Qian (2007).

### Proof

Following standard arguments (Taylor and Karlin 1998; Wilkinson 2018; Higham 2008; Calvetti and Somersalo 2012), we define the probability distribution $p_{i,j,k}(t)=\Pr \left( {\textbf{N}}(t)=(i,j,k)\right)$, where $i,j,k\in \{0,1,2,\dots \}$. (We set $p_{i,j,k}\equiv 0$ if $i<0$, $j<0$ or $k<0$.) Recall from the definition of the GV model (Eq. 6–10) that $b,\Omega >0$ and $\alpha ,\beta \ge 0$. The distribution $p_{i,j,k}$ obeys an evolution equation, or discrete master equation, of the form

B1 $$\begin{aligned} \frac{dp_{i,j,k}}{dt}= & {} b\left[ (i-1)p_{i-1,j,k}+(j-1)p_{i,j-1,k}+(k-1)p_{i,j,k-1}\right] \nonumber \\{} & {} +d\left[ (i+1)p_{i+1,j,k}+(j+1)p_{i,j+1,k}+(k+1)p_{i,j,k+1}\right] \nonumber \\{} & {} +(i+1)\left( \frac{i}{\Omega }+\frac{\alpha }{\Omega }j+\frac{\beta }{\Omega }k\right) p_{i+1,j,k} + (j+1)\left( \frac{\beta }{\Omega }i+\frac{j}{\Omega }+\frac{\alpha }{\Omega }k\right) p_{i,j+1,k}\nonumber \\{} & {} +(k+1)\left( \frac{\alpha }{\Omega }i+\frac{\beta }{\Omega }j+\frac{k}{\Omega }\right) p_{i,j,k+1} - (b+d)(i+j+k)p_{i,j,k}\nonumber \\{} & {} -\left[ i\left( \frac{i-1}{\Omega }+\frac{\alpha }{\Omega }j+\frac{\beta }{\Omega }k\right) +j\left( \frac{\beta }{\Omega }i+\frac{j-1}{\Omega }+\frac{\alpha }{\Omega }\right) +k\left( \frac{\alpha }{\beta }i+\frac{\beta }{\Omega }j+\frac{k-1}{\Omega }\right) \right] p_{i,j,k}.\nonumber \\ \end{aligned}$$

**Existence**: Setting $p_{0,0,0}=1$ and all other $p_{i,j,k}=0$ satisfies the equilibrium condition $\frac{dp_{i,j,k}}{dt}=0$ for all *i*, *j*, *k*, by inspection.

**Uniqueness**: Suppose $\frac{dp_{i,j,k}}{dt}=0$ for all *i*, *j*, *k*. It follows immediately from Eq. (B1) that

$$\begin{aligned} \frac{dp_{0,0,0}}{dt}={}&d\left[ p_{1,0,0}+p_{0,1,0}+p_{0,0,1}\right] =0.\\ \implies {}&p_{1,0,0}=p_{0,1,0}=p_{0,0,1}\equiv 0.\\ \frac{dp_{1,0,0}}{dt}={}&d\left[ 2p_{2,0,0}+p_{1,1,0}+p_{1,0,1}\right] +\left( \frac{2}{\Omega }\right) p_{2,0,0}=0.\\ \implies {}&p_{2,0,0}=p_{1,1,0}=p_{1,0,1}\equiv 0.\\ \frac{dp_{1,1,0}}{dt}={}&d\left[ 2p_{2,1,0}+2p_{1,2,0}+p_{1,1,1}\right] +2\left( \frac{1+\alpha }{\Omega }\right) p_{2,1,0}+2\left( \frac{\beta +1}{\Omega }\right) p_{1,2,0}=0.\\ \implies {}&p_{2,1,0}=p_{1,2,0}=p_{1,1,1}\equiv 0.\\ \vdots {}&\end{aligned}$$

Continuing iteratively, it is clear that $\pi ({\textbf{N}})=0$ whenever ${\textbf{N}}\ne 0$, while $p_{0,0,0}$ is not so constrained. Normalization of the distribution enforces $\pi ({\textbf{N}})=\delta ({\textbf{N}})$. $\square$

## Appendix C: Proof of proposition 2

Following Anderson and Kurtz (2015), we summarize the elements of a chemical reaction network. The network comprises a set of *m* species ${\mathcal {S}}$ (in our case, ${\mathcal {S}}=\{N_1,N_2,N_3\}$), a set of complexes ${\mathcal {C}}$, which are nonnegative integer linear combinations of species (for example, $N_1+N_2$ is the complex $y=(1,1,0)$; $2N_1$ is the complex $y'=(2,0,0)$, etc.), and a finite set ${\mathcal {R}}$ of reactions (e.g. reaction 1 might be $N_1+N_2\rightarrow 2N_2$). A reaction network of this form has $\ell$ linkage classes, which are the number of connected components of the reaction network graph. We index the reactions $1,\dots ,k,\dots ,\vert {\mathcal {R}}\vert$. In a deterministic mass-action kinetics model, the reaction taking complex *y* to complex $y'$ has rate $\kappa c^y,$ where $c\in {\mathbb {R}}^m_+$ is the vector of species concentrations, $\kappa$ is a molecular rate constant, and $c^y=\prod _{i=1}^mc_i^{y_i}$. An equilibrium concentration $c_*$ for a deterministic network is “complex-balanced" if for every complex $\eta \in {\mathcal {C}}$, the net production and consumption rates of $\eta$ are equal, i.e.

C2 $$\begin{aligned} \sum _{\{k:\eta =y_k\}}\kappa _kc^{y_k}=\sum _{\{k:\eta =y'_k\}}\kappa _kc^{y'_k} \end{aligned}$$

where the LHS sums over source complexes and the RHS sums over product complexes. Anderson and Kurtz (2015) further define a chemical reaction network $\{{\mathcal {S}},{\mathcal {C}},{\mathcal {R}}\}$ to be *weakly reversible* if for any reaction $y_k\rightarrow y'_k \in {\mathcal {R}},$ there is a finite sequence of reactions beginning with $y'_k$ as a source complex and ending with $y_k$ as a product complex, e.g. $y'_k\rightarrow y_1\rightarrow y_2\rightarrow \ldots \rightarrow y_r\rightarrow y_k$.

With this background, we are able to prove Proposition 2:

*Let* ${\textbf{N}}=(N_1,N_2,N_3)$ *be the population vector of the minimal model Eqs.* (11–14). *The reaction system Eqs.* (11–14) *has four distinct stationary distributions. Three may be expressed as component-wise stationary distributions of the form*

$$\begin{aligned} \pi (N_i)=\frac{(r\Omega )^{N_i}}{N_i!(e^{r\Omega }-1)}\delta (N_{i+1})\delta (N_{i+2}), \end{aligned}$$

*for* $i\in \{1,2,3\}$, $N_i\ge 1$, *with* $\delta (x)$ *being the distribution with unit probability at* $x=0$, *and with index addition taken cyclically on* $\{1,2,3\}$. *The fourth is* $\pi ({\textbf{N}})=\delta ({\textbf{N}})$*.*

### Proof

The distribution $\pi ({\textbf{N}})=\prod _{i=1}^3\delta (N_i)$, which represents complete extinction, is clearly an invariant distribution for the system Eqs. (11–14). It remains to show that the only other stationary distributions have the form Eq. (15).

We start by showing that neither the full three-dimensional system nor the two-dimensional subsystem admit complex balanced equilibria, while each one-dimensional subsystem does admit a complex balanced equilibrium (CBE). Without loss of generality, we will set $i=1$; the remaining stationary distribution follows by permutation of indices. First, we consider the full three-dimensional system. We write the reaction network from Eq. (11)–(14) in the more compact form

C3 $$\begin{aligned}{} & {} N_1\rightleftharpoons 2N_1 \qquad N_2\rightleftharpoons 2N_2 \qquad N_3\rightleftharpoons 2N_3 \nonumber \\{} & {} N_1\leftarrow N_1+N_2\rightarrow N_2 \quad N_1\leftarrow N_1+N_3\rightarrow N_3 \quad N_2\leftarrow N_2+N_3\rightarrow N_3\qquad \end{aligned}$$

The system has nine complexes: ${\mathcal {C}}=\{N_1,N_2,N_3,2N_1,2N_2,2N_3,N_1+N_2,N_1+N_3,N_2+N_3\}$ and six linkage classes $\ell$ (the distinct connected components of the reaction network, displayed in Eq. (C3)). The stoichiometric reaction vectors, representing the change in number of each species that results from each reaction, span the entire space of dimension $s=3$. The *network deficiency* is $\delta :=\vert {\mathcal {C}}\vert -\ell -s$; for our system $\delta =9-6-3=0$. The complex balanced equilibrium theorem (Anderson et al. 2010; Anderson and Kurtz 2015) establishes that a zero-deficiency network has a CBE if and only if it is weakly reversible. The minimal reaction network Eq. (C3) is not weakly reversible. For example, the complex $y=N_1+N_2$ appears as the source complex in the reaction $N_1+N_2\rightarrow N_1$, but there is no reaction path leading from the product complex $y'=N_1$ back to *y*. We conclude that the network does not admit a CBE. Consequently, there is no stationary distribution in which all three species have nonzero populations. (Failure of weak reversibility coincides with the intuition that once the population enters a two-dimensional subspace on a coordinate plane, there is no reaction to bring the system back into the full three-dimensional space.)

Next suppose, again WLOG, that $N_3$ is the first population to go extinct. The two dimensional subsystem $(N_1,N_2,0)$ is an absorbing set, within which the system has the reduced reaction network

$$\begin{aligned}{} & {} N_1\rightleftharpoons 2N_1 \qquad N_2\rightleftharpoons 2N_2 \\{} & {} N_1\leftarrow N_1+N_2\rightarrow N_2 \end{aligned}$$

This reaction network has deficiency $\delta =5-3-2=0$. Invoking the complex balanced equilibrium theorem again, since this two-dimensional network also fails to be weakly reversible, it again does not admit a stationary distribution.

Within the $(N_1,N_2,0)$ subsystem, either species could go extinct. Suppose (again WLOG) that $N_2$ goes extinct next. Now the network reduces to the one-dimensional subsystem

C4 $$\begin{aligned} N_1\rightleftharpoons 2N_1 \end{aligned}$$

This reaction network also has zero deficiency, $\delta =2-1-1=0$, but unlike the previous cases, it is weakly reversible. In this case, the stationary distribution theorem (Anderson et al. 2010) establishes that the subsystem Eq. (C4) has a unique stationary distribution which is a truncated Poisson distribution:

C5 $$\begin{aligned} \pi (N_1)=\frac{(r\Omega )^{N_1}}{N_1!\left( e^{r\Omega }-1\right) }, \ N_1\in {\mathbb {N}}. \end{aligned}$$

The structure of the three-population minimal reaction network Eq. (11–14) is invariant under permutation of the indices $\{1,2,3\}$. Therefore, with system size $\Omega$, system Eq. (11–14) admits precisely three non-degenerate stationary distributions, namely $\pi (N_i)\delta (N_{i+1})\delta (N_{i+2})$, where $\delta (N)$ is the distribution with unit probability at $n=0$, and indicial addition is taken cyclically on $\{1,2,3\}$. This completes the proof of Proposition 2. $\square$

### Remark

Note that our proof of Proposition 2 also shows that neither the full three-dimensional system (i.e., the system where no species have gone extinct) nor any two-dimensional subsystem (i.e., the system where exactly one species has gone extinct) admit any equilibria. Furthermore, the complex balanced equilibrium theorem establishes uniqueness of stationary solutions on each irreducible partition of the state space. Taken together, these two observations imply that the four stationary distributions described in Proposition 2 are the only possible stationary distributions for the system.

## Appendix D: Formulation of discrete first-passage problems

To construct the first-passage location and first-passage time problems for the stochastic minimal model as described in Sect. 4, we first derive the infinitesimal generator matrix ${\mathcal {L}}$, with appropriate boundary conditions. To track when a particular species goes extinct, we require the coordinate planes $N_i=0$ to be absorbing, where $i\in \{1,2,3\}$. While in principle the populations in the minimal model are unbounded, we truncate the state space and obtain a finite-dimensional operator ${\mathcal {L}}$. To enforce conservation of probability, we impose reflecting boundary conditions on the planes $N_i=A$. The dimension of the resulting operator ${\mathcal {L}}$ is $(A+1)^3\times (A+1)^3$. We found that setting $A=2\Omega$ maintained a reasonable balance between accuracy and computational efficiency.

Given the stochastic reaction net for the minimal model in Eqs. (11–14) and the boundary conditions specified above, we construct ${\mathcal {L}}$ following Taylor and Karlin (1998); Higham (2008); Wilkinson (2018). Each entry of ${\mathcal {L}}$ corresponds to a particular pair of states in the discrete system. Consider a state *i* with population vector ${\textbf{N}}=(N_1,N_2,N_3)$. If *i* is not on an absorbing boundary, the corresponding row of ${\mathcal {L}}$ is given by

D6 $$\begin{aligned}{} & {} \left[ {\mathcal {L}}\right] _{i,i-(1+2\Omega )^2}=\frac{\alpha }{\Omega }N_1N_3+\frac{\beta }{\Omega }N_2N_3+\frac{N_3(N_3-1)}{\Omega }, \end{aligned}$$

D7 $$\begin{aligned}{} & {} \left[ {\mathcal {L}}\right] _{i,i-(1+2\Omega )}=\frac{N_1(N_1-1)}{\Omega }+\frac{\alpha }{\Omega }N_1N_2+\frac{\beta }{\Omega }N_1N_3,\end{aligned}$$

D8 $$\begin{aligned}{} & {} \left[ {\mathcal {L}}\right] _{i,i-1}=\frac{\beta }{\Omega }N_1N_2+\frac{N_2(N_2-1)}{\Omega }+\frac{\alpha }{\Omega }N_2N_3,\end{aligned}$$

D9 $$\begin{aligned}{} & {} \left[ {\mathcal {L}}\right] _{i,i+1}=rN_2\mathbbm {1}_{\{N_2<2\Omega \}}(N_2),\end{aligned}$$

D10 $$\begin{aligned}{} & {} \left[ {\mathcal {L}}\right] _{i,i+(1+2\Omega )}=rN_1\mathbbm {1}_{\{N_1<2\Omega \}}(N_1),\end{aligned}$$

D11 $$\begin{aligned}{} & {} \left[ {\mathcal {L}}\right] _{i,i+(1+2\Omega )^2}=rN_3\mathbbm {1}_{\{N_3<2\Omega \}}(N_3),\end{aligned}$$

D12 $$\begin{aligned}{} & {} \left[ {\mathcal {L}}\right] _{i,i}=-\sum _{j\ne i}\left[ {\mathcal {L}}\right] _{i,j}. \end{aligned}$$

In Eqs. (D9 – D11), $\mathbbm {1}_{A}(X)$ is an indicator function that equals to unity if $X\in A$ and is zero otherwise. This indicator function enforces the reflecting boundary if *i* is on one of the specified planes. If *i* is on one of the absorbing boundaries, then the corresponding row of ${\mathcal {L}}$ is simply given by

D13 $$\begin{aligned} \left[ {\mathcal {L}}\right] _{i,i}=1. \end{aligned}$$

We index ${\mathcal {L}}$ in the method outlined above so that our implementation is compatible with meshgrid in MATLAB; see https://github.com/nwbarendregt/StochasticHC. By constructing ${\mathcal {L}}$ as specified above, we can formulate both the first-passage location problem in Eq. (16) and the first-passage time problem in Eq. (17) by modifying the right-hand side of the equation.
